# The Importance of Tumor–Host Interactions in Adult B-Cell Leukemias and Lymphomas

**DOI:** 10.3390/ijms21186915

**Published:** 2020-09-21

**Authors:** Silvia Deaglio, Tanja Nicole Hartmann

**Affiliations:** 1 Department of Medical Sciences, University of Turin, 10126 Turin, Italy; 2 Department of Internal Medicine I, Medical Center and Faculty of Medicine, University of Freiburg, 79106 Freiburg, Germany

The tumor microenvironment plays a crucial role in driving the behavior and the aggressiveness of neoplastic cells. Over the years, the complex network of tumor–host interactions has been studied in detail to gain insights about its contribution to disease evolution and progression. In the context of B-cell leukemias and lymphomas, the lymphoid niche provides optimal conditions to cancer cells and sustains their survival, drug resistance and immune escape through a delicate interplay of soluble factors and cell–cell interactions. Furthermore, it is now widely accepted that malignant B cells shape their environment to educate the surrounding cells towards tolerance and tumor support. This issue of IJMS is fully dedicated to the unraveling of these mechanisms in leukemia and lymphoma.

Mangolini and Ringshausen drive our attention to the bidirectional relationship between malignant B lymphocytes and stromal cells. They effectively outline the derailment of intracellular signaling, gene expression and metabolic adaptation and the consequences on leukemic cells’ behavior and survival. The authors clarify that reciprocal interactions in the microenvironment can occur through multiple mediators, including not only soluble factors and membrane-bound proteins but also vesicle exchange and membrane extensions called tunneling nanotubes, promoting cell–cell physical interaction [1]. By exploiting different strategies, malignant B lymphocytes and the surrounding non-tumor cells can exchange a number of molecules and factors, such as miRNAs that in turn affect global gene expression and biological features of malignant cells [2]. Importantly, reciprocal interactions also influence stromal cells biology, and understanding the molecular mechanisms behind this bidirectional communication can highlight potential vulnerabilities that can be exploited from a therapeutic perspective [1].

Chemokines and their receptors are one of the best-characterized molecular features of B cell leukemia and lymphoma communication with the microenvironment. Among them, the CXCR4–CXCL12 axis is known to regulate the trafficking to and from the lymphoid niche in different B-cell malignancies. In this Issue, Pansy and colleagues provide experimental evidence of the association of CXCR4 overexpression and poor clinical outcome in a cohort of diffuse large B cell lymphoma (DLBCL) patients, supporting the view that CXCR4 levels can be of prognostic relevance. Molecules interrupting this signaling may be therapeutically exploited to induce apoptosis of DLBCL cells [3].

Similarly to chemokines, integrin signaling, such as that occurring through VLA-4, has a longstanding track record as a key regulator of organ infiltration by controlling cell adhesion and homing, as thoroughly described by Härzschel and colleagues with a dedicated review included in this Issue [4]. The biological role of VLA-4 in positioning and retaining cells in particular niches thereby explains the prognostic and predictive role of its subunit CD49d as a robust biomarker for high risk in chronic lymphocytic leukemia (CLL). 

A further piece in the puzzle of tumor–host interactions that is gaining progressive relevance is that of metabolic changes occurring within the niche. Metabolic adaptation to local environmental conditions is a feature of both transformed and non-transformed cells and can lead to or exacerbate specific dysfunctions, having the immune response as one of the most central targets. Growing interest in this topic is witnessed in this Special Issue by the review from Beielsten et al. who provide an overview of the main metabolic changes observed in B cell malignancies and their effects on different populations of non-transformed cells residing in the lymphoid niche. There, alteration of key metabolic processes such as glycolysis or amino acid catabolism can affect the phenotype and functions of cancer-associated fibroblasts, macrophages and T cells overall promoting tumor survival and immune escape and therefore representing an appealing therapeutic target [5].

The topic of immunomodulation and tumor escape from immune surveillance is further addressed in the Issue by different reviews focusing on the molecular mechanisms and pathways driving immune response dysfunctions [6], on the impact of small inhibitors currently in the clinical practice [7], on the involvement of specific cellular players and the possibility to engage them for immunotherapy strategies [8], and on the efforts to design novel therapeutic approaches for B cell malignancies targeting immune and inflammatory cells [9].

Lastly, the importance of the tumor microenvironment in B-cell neoplasia is technically reflected by the difficulties of xenografting primary malignant B cells into murine models unless provided with accessory non-transformed cells to recapitulate the host niche. In this sense, the research article by Decker et al. in this Special Issue can be instrumental to the generation of new in vivo models as important tools in the study of B-cell leukemias and lymphoma biology and therapy [10].

## References

[1] Mangolini M., Ringshausen I. (2020). Bone Marrow Stromal Cells Drive Key Hallmarks of B Cell Malignancies. Int. J. Mol. Sci..

[2] El-Daly S.M., Bayraktar R., Anfossi S., Calin G.A. (2020). The Interplay between MicroRNAs and the Components of the Tumor Microenvironment in B-Cell Malignancies. Int. J. Mol. Sci..

[3] Pansy K., Feichtinger J., Ehall B., Uhl B., Sedej M., Roula D., Pursche B., Wolf A., Zoidl M., Steinbauer E. (2019). The CXCR4-CXCL12-Axis Is of Prognostic Relevance in DLBCL and Its Antagonists Exert Pro-Apoptotic Effects In Vitro. Int. J. Mol. Sci..

[4] Harzschel A., Zucchetto A., Gattei V., Hartmann T.N. (2020). VLA-4 Expression and Activation in B Cell Malignancies: Functional and Clinical Aspects. Int. J. Mol. Sci..

[5] Beielstein A.C., Pallasch C.P. (2019). Tumor Metabolism as a Regulator of Tumor-Host Interactions in the B-Cell Lymphoma Microenvironment-Fueling Progression and Novel Brakes for Therapy. Int. J. Mol. Sci..

[6] Arruga F., Gyau B.B., Iannello A., Vitale N., Vaisitti T., Deaglio S. (2020). Immune Response Dysfunction in Chronic Lymphocytic Leukemia: Dissecting Molecular Mechanisms and Microenvironmental Conditions. Int. J. Mol. Sci..

[7] Mhibik M., Wiestner A., Sun C. (2019). Harnessing the Effects of BTKi on T Cells for Effective Immunotherapy against CLL. Int. J. Mol. Sci..

[8] Hofland T., Eldering E., Kater A.P., Tonino S.H. (2019). Engaging Cytotoxic T and NK Cells for Immunotherapy in Chronic Lymphocytic Leukemia. Int. J. Mol. Sci..

[9] Calabretta E., d’Amore F., Carlo-Stella C. (2019). Immune and Inflammatory Cells of the Tumor Microenvironment Represent Novel Therapeutic Targets in Classical Hodgkin Lymphoma. Int. J. Mol. Sci..

[10] Decker S., Zwick A., Khaja Saleem S., Kissel S., Rettig A., Aumann K., Dierks C. (2019). Optimized Xenograft Protocol for Chronic Lymphocytic Leukemia Results in High Engraftment Efficiency for All CLL Subgroups. Int. J. Mol. Sci..

